# Complete Genome Sequence of a Putative Densovirus Infecting the Carrot Psyllid *Bactericera trigonica*

**DOI:** 10.1128/MRA.01103-19

**Published:** 2019-11-27

**Authors:** Saptarshi Ghosh, Noa Sela, Murad Ghanim

**Affiliations:** a Department of Entomology, Volcani Center, Rishon LeZion, Israel; Portland State University

## Abstract

Here, we report the genome of a putative densovirus infecting the carrot psyllid Bactericera trigonica, obtained by inverse PCR and named *Bactericera trigonica* densovirus (BtDNV). The ambisense genome of BtDNV is identical in structure to those of the ambidensoviruses, and its encoded proteins share the highest sequence identity with the Asian citrus psyllid *Diaphorina citri* densovirus.

## ANNOUNCEMENT

The carrot psyllid Bactericera trigonica transmits “*Candidatus* Liberibacter solanacearum,” which causes major economic losses (1). Introduction of native recombinant viruses as expression vectors inside their insect vectors could be an alternative management strategy for disruption of “*Ca*. Liberibacter solanacearum” transmission. Ambidensoviruses (Parvoviridae, Densovirinae) are linear single-stranded DNA (ssDNA) viruses with an ambisense genome expression strategy that infect insects, crustaceans, and echinoderms (2). The autoreplicative nature and small (5- to 6-kb) genome length of densoviruses facilitate their use as vectors for gene delivery (3, 4). Here, we describe the complete genome of a putative densovirus infecting B. trigonica, which has the potential for development as an expression vector. Total RNA was extracted from *B. trigonica* adults collected in Israel (5), and mRNA was enriched by oligo(dT). The mRNA was reverse transcribed by random hexamers and sequenced with an Illumina HiSeq 4000 instrument. Illumina transcriptome sequencing (RNA-seq) reads were *de novo* assembled using the Trinity platform, as decribed by Ghosh et al. (5), and used to search for viral proteins at the NCBI nonredundant protein (nr) database, as previously described (6), using Diamond BLASTX with the command line “diamond blastx -query trinity_file -db nr_database.dmnd -out outputfile.xml -outfmt 5 -salltitles.” We filtered for the sequences that have an E value smaller than 1E−5. Subsequently, we found several contigs that matched *Diaphorina citri* densovirus with an E value smaller than 1E−10. The search revealed the presence of nine short contigs with similarity to densoviruses. However, the short contigs assembled from the cDNA library varied, and no consensus sequence could be derived. Therefore, primers were designed from one of the nine contigs to PCR amplify a fragment encoding the structural proteins. PCR amplification (primers F-5′-CCAATATGCAATTGAGGTTGTG-3′ and R-5′-TTGGTTTGAACAACAAATTGCATA-3′) and Sanger sequencing of a 1,631-bp fragment of the densovirus from DNA extracted from *B. trigonica* adults by using a DNeasy blood and tissue kit (Qiagen) confirmed its presence. The complete genome was obtained by PCR amplification of the replicative forms of the virus using an inverse PCR strategy with primers (F-5′-GAACTGAGCTCGAGCACGAGCATC-3′ and R-5′-TACGGGCTCAGTGGGAGGTGAAC-3′) designed to amplify a 3,124-bp fragment, followed by primer walking from both directions. We tentatively name this virus *Bactericera trigonica* densovirus (BtDNV).

The BtDNV genome is comprised of 4,631 nucleotides (47% GC content), starting and terminating with 90- and 101-bp-long 5′ and 3′ untranscribed region (UTR) regions, respectively, and it contains four open reading frames (ORFs). The ORFs were identified using the ORFfinder and CDD search tools (NCBI). The BtDNV genome organization facilitates bidirectional transcription similar to that of ambidensoviruses, with the nonstructural (NS) proteins (ORF1 and ORF2) being translated from the sense strand and the structural proteins (ORF3 and ORF4) being translated from the complementary strand. BtDNV-encoded proteins were compared and analyzed using the pBLAST algorithm. ORF1 (nucleotides 90 to 1439) initiated at a TTG start codon and encodes a 449-amino acid (aa) nonstructural protein with highest identity (34.7%) to a nonstructural protein of *Diaphorina citri* densovirus (DcDNV; GenBank accession number ANH56808) (7). A nonstructural protein similar (44.6%) to that of the NS1 protein of DcDNV (ANH56809), putatively initiated at a CTG start codon, is translated from ORF2 (nucleotides 182 to 2212) of BtDNV. Similarly to DcDNV, NS2- or NS3-translating ORFs were also found to be absent in the BtDNV genome. The structural proteins VP4 (573 aa) and VP1 (202 aa), encoded in ORF3 (nucleotides 3941 to 2220) and ORF4 (nucleotides 4536 to 3928), had sequence identity with the VP4 (44.6%) and VP1 proteins (39.1%) of DcDNV, respectively. An 8-bp-long intergenic region separates the ORFs on the sense strand from those on the complementary strand.

### Data availability.

The BtDNV genome is available in GenBank under accession number MN364694.

## References

[1] Ghanim M, Achor D, Ghosh S, Kontsedalov S, Lebedev G, Levy A 2017 “*Candidatus* Liberibacter asiaticus” accumulates inside endoplasmic reticulum associated vacuoles in the gut cells of *Diaphorina citri*. Sci Rep 7:16945. doi:10.1038/s41598-017-16095-w. 29208900 PMC5717136

[2] Cotmore SF, Agbandje-McKenna M, Canuti M, Chiorini JA, Eis-Hubinger A-M, Hughes J, Mietzsch M, Modha S, Ogliastro M, Pénzes JJ 2019 ICTV virus taxonomy profile: *Parvoviridae*. J Gen Virol 100:367–368. doi:10.1099/jgv.0.001212. 30672729 PMC6537627

[3] Royer C, Bossin H, Romane C, Bergoin M, Couble P 2001 High amplification of a densovirus-derived vector in larval and adult tissues of *Drosophila*. Insect Mol Biol 10:275–280. doi:10.1046/j.1365-2583.2001.00264.x. 11437919

[4] Hu L, Zhang L, Shen C, Lu J, Zhang J, Hu Y 2007 The densovirus of *Periplaneta fuliginosa* (PfDNV) as an insect vector for persistent foreign gene expression *in vivo*. Biochem Biophys Res Commun 358:976–982. doi:10.1016/j.bbrc.2007.04.210. 17517375

[5] Ghosh S, Jassar O, Kontsedalov S, Lebedev G, Wang C, Turner D, Levy A, Ghanim M 2019 A transcriptomics approach reveals putative interaction of *Candidatus* Liberibacter solanacearum with the endoplasmic reticulum of its psyllid vector. Insects 10:279. doi:10.3390/insects10090279. PMC6780682 31480697

[6] Ghosh S, Kanakala S, Lebedev G, Kontsedalov S, Silverman D, Alon T, Mor N, Sela N, Luria N, Dombrovsky A, Mawassi M, Haviv S, Czosnek H, Ghanim M 2019 Transmission of a new polerovirus infecting pepper by the whitefly *Bemisia tabaci*. J Virol 93:e00488-19. doi:10.1128/JVI.00488-19. 31092571 PMC6639281

[7] Nigg JC, Nouri S, Falk BW 2016 Complete genome sequence of a putative densovirus of the Asian citrus psyllid, *Diaphorina citri*. Genome Announc 4:e00589-16. doi:10.1128/genomeA.00589-16. 27469948 PMC4966452

